# Cystic Lymphangioma: Are Triglycerides Always Measurable?

**DOI:** 10.1155/2018/9591420

**Published:** 2018-03-01

**Authors:** Silke François, Manuella Martin, Olivier Costa, Daniel Urbain, Fazia Mana

**Affiliations:** ^1^Universitair Ziekenhuis Brussel (UZ Brussel), Vrije Universiteit Brussel (VUB), Department of Gastro-Enterology, Laarbeeklaan 101, 1090 Brussels, Belgium; ^2^Universitair Ziekenhuis Brussel (UZ Brussel), Department of Clinical Biology, Laarbeeklaan 101, 1090 Brussels, Belgium

## Abstract

The presence of chylous fluid with high triglycerides levels on endoscopic ultrasound- (EUS-) guided fine needle aspiration (FNA) is very pathognomonic for the diagnosis of cystic lymphangiomas of the pancreas. In our case report the puncture of the pancreatic cyst showed a typical milky fluid though measurable triglyceride concentrations were absent in the laboratory. Two possible explanations were found. First of all grossly lipemic samples show a slower rate of color development than do clear serums which can produce a false negative result if the sample is insufficiently diluted. Secondly, high lipase levels can divide triglycerides in glycerol and fatty acids, making the concentration of triglycerides undetectable.

## 1. Introduction

Cystic lymphangiomas are benign cystic tumors which arise from the lymphatic system due to blockage of the lymphatic flow which can be congenital or due to inflammation, trauma, or cancer. Lymphangiomas occur mostly in children, especially found in the neck (75%) and axillae (20%) but they can be found anywhere in the body [1, 2]. Although diagnosis is based on histopathology, the presence of chylous fluid with high triglycerides levels on endoscopic ultrasound-guided fine needle aspiration (EUS-FNA) is very pathognomonic [3]. Lymphangiomas of the pancreas are very rare and present a challenge concerning differential diagnosis with other fluid-containing masses, benign as well as malign, especially when the fluid is not “typical.”

We present the case of a young woman with a pancreatic cyst where EUS-FNA showed a typical milky fluid but without measurable triglyceride concentrations. In this case report we try to explain this atypical biochemical finding.

## 2. Case Report

**Figure 1:**
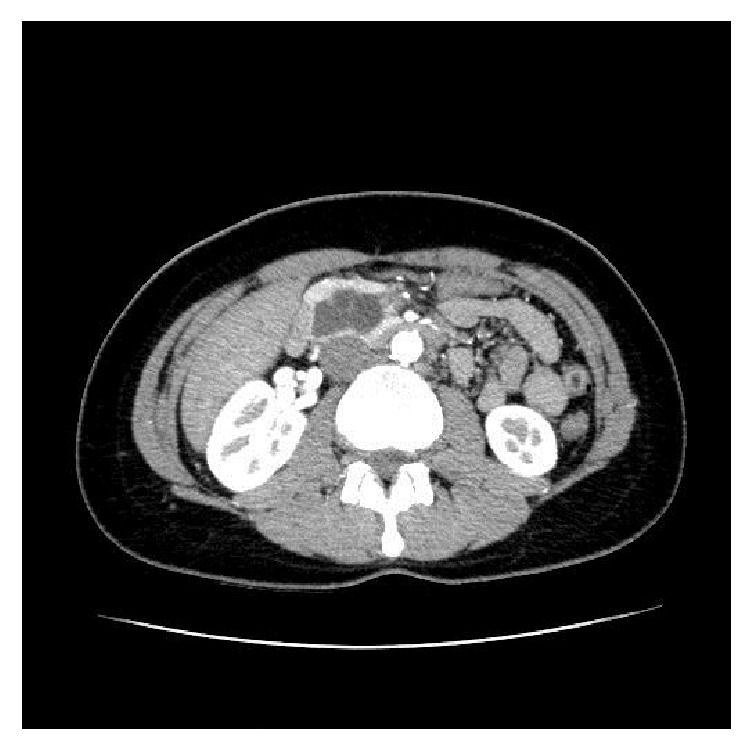
Abdominal CT scan showing a large cystic lesion in the head of the pancreas.

A 29-year-old female was evaluated for mild episodes of chronic pain in the right hypochondriac region which worsened for a few days. She also complained about constipation and mild dyspepsia. Medical history showed a scoliosis and exercise-induced asthma. Physical examination did not reveal any abnormalities. Routine laboratory tests were unremarkable. An ultrasound of the abdomen was performed and showed a cystic lesion (diameter 5.6 cm), adjacent to the gall bladder. Subsequently, a contrast-enhanced computer tomographic scan and MRI were performed and showed a large multiloculated cyst (28 × 33 × 46 mm) with multiple thin septa in the head and neck of the pancreas (Figure 1).

**Figure 2:**
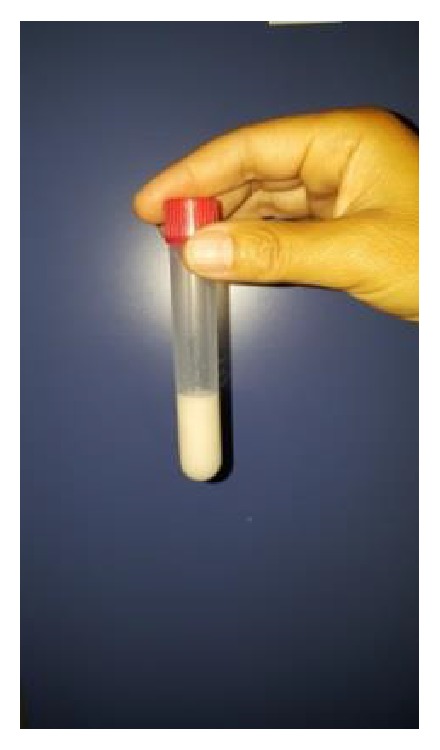
Sample showing the milky aspect of the aspirated fluid from the cystic lesion.

An endoscopic ultrasound (EUS) was performed and confirmed the presence of a large multiloculated cyst (5 × 5 cm) with clear borders, without nodules, calcifications, or perilesional lymphadenopathy. There were micro- and macrocystic components with thin septa and a clear anechoic content. The remaining pancreatic parenchyma was normal. EUS-guided aspiration with a 22G needle demonstrated chylous liquid (Figure 2) but analyses showed low triglyceride (TG) levels (<10 mg/dl) (Table 1). Due to this discrepancy, a second EUS-FNA was performed three months later, again showing a milky fluid aspect. The biochemical evaluation of the cystic fluid again revealed low triglycerides (<10 mg/dl) (Table 1).

Cytological examination showed a hypocellular monster with only rare lymphocytes and neutrophils. There were no epithelial cells. There were no arguments for malignancy.

The diagnosis of a cystic lymphangioma was withheld based on the macroscopic chylous aspect of the fluid, cytological examination, and negative CEA measurement. A conservative policy was agreed on with a follow-up by MRI within 6 months.

## 3. Discussion

Cystic lymphangiomas are rare multiloculated soft cystic masses, with flat endothelial cells that line the cyst wall. The cysts are variable in size and composed of dilated lymphatic channels that are divided by thin septa [1, 2]. Pancreatic lymphangiomas are considered to be of pancreatic origin if they are within the parenchyma, adjacent to the pancreas or connected to it by a pedicle. They are extremely rare, accounting for less than 1% of all lymphangiomas and about 0.2% of pancreatic neoplasms [3, 4]. They are most commonly found in women and mostly located in the body and tail of the pancreas [5, 6].

Pancreatic cystic tumors are a diagnostic challenge because conventional imaging studies cannot characterize most lesion with sufficient certainty. The differential diagnosis of a cystic pancreatic lesion is broad including serous and mucinous cystadenoma, mucinous cystadenocarcinoma, pseudocysts, intraductal papillary mucinous tumors (IPMT), cystic neuroendocrine tumors (NET), solid pseudopapillary tumor, parasitic cysts, or necrosis/cystic degeneration of ductal carcinoma [3, 7, 8]. The most important thing in the clinical decision making is to be sure of the nonmalignant potential of the cystic lesion. EUS-FNA (cytology, amylase, and CEA) was more sensitive (76%) than CT or MRI (resp., 48% and 34%) for differentiating neoplastic from nonneoplastic cysts [9]. A pooled analysis published in 2005 shows that a CEA < 5 ng/ml suggests a benign pancreatic cystic lesion (serous cystadenoma or pseudocyst) with a specificity of 95%, a sensitivity of 50%, and a positive predictive value of 94%. This implies that despite a CEA < 5 ng/ml, 6% of the cystic lesions will be mucinous cystadenoma of mucinous cystadenocarcinoma with a premalignant of malignant character [8]. Therefore, a close follow-up program by medical imaging is recommended, even if the CEA level is considered to be negative. It is suggested that a CEA > 5 ng/ml in the cyst fluid may suggest the presence of a premalignancy or malignancy [8].

As lymphangiomas are benign tumors without malign potential, treatment depends on the symptoms. In symptomatic patients complete excision is curative but in asymptomatic patients treatment is conservative [6]. This is in contrast with other lesions like IPMT or mucinous cystadenoma where surgery is indicated depending on the location, FNA results, and size of the tumors. In malignant lesions like pseudopapillary tumors surgery is always indicated. Pancreatic surgery may vary from a simple excision to an extensive pancreatic resection, depending on the tumor location.

The diagnosis of lymphangioma of the pancreas has often been established after surgery. However, since the development of EUS-FNA, diagnosis is based on high triglyceride levels alone or on a combination of high triglyceride levels and numerous lymphocytes in the aspirate [5]. Lymphangiomas are in general easily recognized when having a macroscopically milky fluid aspect. However, as in our case, when macroscopic diagnosis is not confirmed by high triglycerides, doubt can occur. The absence of triglycerides in our case can be supported by two possible explanations.

A first possible explanation can be found in the test itself (TRIG, Vitros 4600, Ortho Clinical Diagnostics; enzymatic, colorimetric; glycerol phosphate oxidase/peroxidase). The concentration of triglycerides is based on a coloring reaction in the laboratory. Grossly lipemic samples show a slower rate of color development than do clear serums, producing a false negative result. To prevent this, samples should be sufficiently diluted before testing [10]. In our laboratory the fluid was diluted 1 : 2 before the analysis. Possibly this was insufficient.

Another explanation can be amylase in the fluid. Due to the high levels of amylase we presume a high lipase concentration. A high lipase level will divide triglyceride in glycerol and fatty acids making the concentration of triglycerides undetectable (see (1)). The milky macroscopic aspect is then explained by the obtained free fatty acids.

(1) $$\begin{array}{c} Triglycerides+H_{2}O\overset{Lipase}{\to }Glycerol+Fatty\;\;acids \end{array}$$

One other similar case report was found where the diagnosis of pancreatic cystic lymphangioma is felt to be appropriate despite absence of triglyceride in the aspirated fluid. Based on similar findings in particular the EUS findings, the milky appearance of the fluid, and the negative cytology results, an identical expectant management was proposed to this patient [4].

## 4. Conclusion

Pancreatic lymphangiomas are rare benign tumors. The diagnosis is generally based on the FNA results with macroscopic milky appearance of the fluid with high triglyceride levels. However, milky fluid without high triglyceride levels can be explained by the coloring reaction of the test itself, where grossly lipemic samples show a slower rate of color development than do clear serums. The second reason can be found in the high lipase concentration in the fluid which divides triglycerides in glycerol and fatty acids, making the concentration of triglycerides undetectable.

## Figures and Tables

**Table 1:** Overview of the biochemical aspects of the aspirated fluid.

	EUS-FNA 20/07/2016	EUS-FNA 02/11/2016
Triglyceride (mg/dl)	<10	<10
Amylase (U/L)	578	3788
CEA (*µ*g/L)	1.7	
